# Distinct hemodynamic and functional connectivity features of fatigue in clinically isolated syndrome and multiple sclerosis: accounting for the confounding effect of concurrent depression symptoms

**DOI:** 10.1007/s00234-023-03174-1

**Published:** 2023-06-10

**Authors:** Despina Antypa, Nicholas John Simos, Theodora Panou, Eirini Spyridaki, Antonios Kagialis, Emmanouela Kosteletou, Eleftherios Kavroulakis, Vasileios Mastorodemos, Efrosini Papadaki

**Affiliations:** 1 Department of Psychiatry, School of Medicine, University of Crete, University Hospital of Heraklion, Crete, Greece; 2 Computational Bio-Medicine Laboratory, Institute of Computer Science, Foundation for Research and Technology, Hellas, Heraklion, Crete, Greece; 3 Institute of Applied Mathematics, Foundation for Research and Technology, Hellas, Heraklion, Crete, Greece; 4 Department of Radiology, School of Medicine, University of Crete, University Hospital of Heraklion, Crete, Greece; 5 Department of Neurology, School of Medicine, University of Crete, University Hospital of Heraklion, Crete, Greece

**Keywords:** Clinically isolated syndrome, Relapsing–remitting multiple sclerosis, Fatigue, Depression, Resting-state fMRI, Time shift analysis

## Abstract

**Purpose:**

This study aims to identify common and distinct hemodynamic and functional connectivity (FC) features for self-rated fatigue and depression symptoms in patients with clinically isolated syndrome (CIS) and relapsing–remitting multiple sclerosis (RR-MS).

**Methods:**

Twenty-four CIS, 29 RR-MS patients, and 39 healthy volunteers were examined using resting-state fMRI (rs-fMRI) to obtain whole-brain maps of (i) hemodynamic response patterns (through time shift analysis), (ii) FC (via intrinsic connectivity contrast maps), and (iii) coupling between hemodynamic response patterns and FC. Each regional map was correlated with fatigue scores, controlling for depression, and with depression scores, controlling for fatigue.

**Results:**

In CIS patients, the severity of fatigue was associated with accelerated hemodynamic response in the insula, hyperconnectivity of the superior frontal gyrus, and evidence of reduced hemodynamics–FC coupling in the left amygdala. In contrast, depression severity was associated with accelerated hemodynamic response in the right limbic temporal pole, hypoconnectivity of the anterior cingulate gyrus, and increased hemodynamics–FC coupling in the left amygdala. In RR-MS patients, fatigue was associated with accelerated hemodynamic response in the insula and medial superior frontal cortex, increased functional role of the left amygdala, and hypoconnectivity of the dorsal orbitofrontal cortex, while depression symptom severity was linked to delayed hemodynamic response in the medial superior frontal gyrus; hypoconnectivity of the insula, ventromedial thalamus, dorsolateral prefrontal cortex, and posterior cingulate; and decreased hemodynamics–FC coupling of the medial orbitofrontal cortex.

**Conclusion:**

There are distinct FC and hemodynamic responses, as well as different magnitude and topography of hemodynamic connectivity coupling, associated with fatigue and depression in early and later stages of MS.

**Supplementary Information:**

The online version contains supplementary material available at 10.1007/s00234-023-03174-1.

## Introduction

Multiple sclerosis (MS) is an autoimmune, inflammatory, demyelinating disorder associated with brain structural, hemodynamic, and functional abnormalities [1–7]. Clinically isolated syndrome (CIS) is considered as the earliest stage of MS [8] with early, progressive changes in brain structure and function [3–5, 9–13]. The topography and progression of these structural and functional changes are not uniform, raising the possibility that the pathophysiological substrate of cognitive and emotional symptoms may change along the course of the disease.

Patients with MS frequently report fatigue and depression symptoms [14]. Fatigue is not only the most common, but perhaps the most disabling symptom in MS, with detrimental effects on everyday functionality and quality of life [14, 15]. MS-related fatigue has been associated with higher lesion load and gray matter (GM) atrophy, hemodynamic changes [16], and brain network dysfunction [14, 15]. Depression symptomatology in MS has been related to lower white matter (WM) volume, loss of integrity of the uncinate fasciculus, and lower functional connectivity (FC) between the amygdala and frontal regions [17]. Although fatigue and depression symptoms frequently co-occur, there is some evidence that MS-related fatigue and depression may not necessarily share common brain functional correlates [14, 15, 18, 19].

Patients with CIS are already afflicted by fatigue and depression, typically of milder severity than in relapsing–remitting MS (RR-MS) patients [20, 21]. Although several studies demonstrated a relationship between fatigue and depression symptomatology and brain function alterations in RR-MS patients [15, 18, 19, 22], there is no direct evidence for such a relation in CIS patients. Moreover, previous studies have attempted to control for concurrent effects of fatigue and depression by excluding patients who reported significant depression symptoms [23, 24], with very few studies employing statistical correction for depression symptom severity [25–27].

Resting-state functional MRI (rs-fMRI) can provide measures of neural activity and function, through various indices of regional FC, as indexed by the overall degree of connectivity of a given region with the rest of the brain. Additionally, time shift analysis (TSA) of rs-fMRI time series can contribute complementary information regarding regional cerebral perfusion alterations. This method quantifies hemodynamic transfer speed through the temporal shift of low-frequency blood oxygenation level–dependent (BOLD) signal fluctuations relative to the blood flow in major cerebral veins [28–30]. Substantial shifts indicate local brain hemodynamics comparable to established MR perfusion techniques [28–30]. Hemodynamic lead (indexed by negative TSA values) indicates accelerated venous drainage in a given region, whereas hemodynamic lag (indexed by positive TSA values) reflects decelerated venous drainage. By using this technique, concurrent FC and hemodynamic measurements are achieved, without the necessity of independent functional and perfusion MRI methods, and their coupling (e.g., extent of voxels displaying hemodynamic lag or lead coupled with relatively low or high intrinsic FC, accordingly) is uniquely and accurately determined. This technique has been applied not only to neurological conditions characterized by severe perfusion disturbances, such as stroke [28–32], but, also, in patients with Alzheimer’s dementia or mild cognitive impairment displaying smaller variations of hemodynamic impairment [33]. By using this method in patients with neuropsychiatric systemic lupus erythematosus (NPSLE), anxiety and depression symptom severity was shown to be associated with accelerated hemodynamic response, paralleled by reduced intrinsic connectivity in limbic and frontal regions, respectively [34]. In another study using rs-fMRI and TSA analysis in NPSLE patients, hemodynamic and FC metrics were integrated by computing direct measures of coupling (or conversely, uncoupling) within specific brain regions. Specifically, uncoupling between hemodynamics and connectivity in parietal cortices was significantly associated with worse performance on several memory and visuomotor tasks among NPSLE patients [35]. Such interdependencies between subtle hemodynamic disturbances and regional brain function may reflect compensatory processes that develop regionally, when venous drainage of this region is delayed by several seconds. Given that brain insults in lupus and MS are presumed to have largely distinct pathophysiological substrates, other forms of hemodynamic connectivity interdependencies are worth examining, namely, what happens to FC in MS when venous drainage in a given region is accelerated or decelerated.

The present study examined aberrant FC, hemodynamics, and the degree of their coupling in relation to fatigue and/or depression symptoms in patients with CIS and RR-MS, by using rs-fMRI and TSA analysis. Specifically, whole-brain indices of intrinsic connectivity, hemodynamic lead or lag, and of their coupling were correlated with fatigue scores, after controlling for depression, and with depression scores (excluding fatigue-related questions), after controlling for fatigue. We hypothesized that, firstly, CIS and RR-MS patients differ substantially in terms of pathophysiological correlates of fatigue and depression symptoms, and, secondly, that MS-related fatigue and depression are linked to different hemodynamics and dysregulation of distinct brain networks.

## Methods

### Participants

Twenty-four CIS and 29 RR-MS patients, recruited through the MS epidemiology program project of Crete [36] and fulfilled the clinical and MRI criteria of the international panel on MS [37], were included in the study. The CIS group consisted of 24 consecutive patients who had experienced a single neurological episode, without a recurrence up to the time of the baseline MRI assessment. Diagnosis of the 29 RR-MS patients was confirmed by a specialist neurologist (VM) and based on the criteria for clinically definite MS, with a relapsing–remitting course and mild physical disability. The onset of disease was defined as the year the patient first experienced clear symptoms of MS and illness duration as the time elapsed between disease onset and testing. A relapse was defined as the appearance of new neurological symptoms or a worsening of pre-existing symptoms lasting for at least 24 h and preceded by a period of clinical stability or improvement of at least 30 days. The Expanded Disability Status Scale was further used to assess physical disability due to disease.

The exclusion criteria were (a) history of alcohol or drug abuse, head injury with loss of consciousness, schizophrenia or bipolar disorder, learning disability, or any other neurological disorder, (b) autoimmune and/or immune-mediated diseases and infectious diseases, (c) significant visual impairment and cerebellar manifestation (e.g., gait ataxia, nystagmus, tremor) and upper limb sensorimotor deficits, and (d) non-right handedness. The two groups were comparable on age (*p*=.9), education level (*p*=.1), and sex ratio (*p*=.8, see Table 1). Comparison fMRI data were available for age-, education-, and gender-matched group of healthy volunteers (*n*=39, mean age = 41.2, SD= 15.1 years, 69% females), without history of psychiatric diagnosis. The study was approved by the University of Crete Hospital Research Ethics Committee, and informed written consent was obtained from all participants.

**Table 1:** Demographic and clinical information by participant group

	1. CIS (*n*=24)	2. RR-MS (*n*=29)	3. HC (*n*=39)	1 vs 2	*p* value 1 vs 3	*p* value 2 vs 3
Age (years)	38.3 (8.7)	38.0 (6.1)	41.2 (15.1)	0.9^1^	.2	.15
Education (years)	11.8 (3.1)	13.3 (3.7)	14.1 (2.5)	0.1^1^	.1	.5
Females	20 (83.3%)	23 (79.3%)	27 (69%)	0.8^2^	.3	.8
EDSS	1.3 (0.7)	3.1 (1.4)	--	<0.001^4^	--	--
(Range)	0–2.5	0.5–6.0	--	--	--	--
Illness duration (years)	4.3 (2.9)	8.2 (5.6)	--	0.002^1^	--	--
(Range)	1–8	1–15	--	--	--	--
Number of relapses	0	3.6 (3.7)	--	<0.001^4^	--	--
(Range)	-	0-14	--	--	--	--
Total IC volume (cm^3^)	1333.7 (146.4)	1356.4 (123.4)	1387.5 (140.4)	0.5^1^	.2	.4
Total GM volume (cm^3^)	668.0 (83.0)	659.9 (62.1)	684.1 (60.2)	0.5^3^	.3	.1
Total WM volume (cm^3^)	503.8 (61.0)	471.9 (70.5)	499.5 (43.3)	0.07^3^	.5	0.06
Total lesion volume (cm^3^)	4.5 (6.1)	11.6 (15.9)	--	0.03^3^	--	--

*EDSS* expanded disability status scale, *HC* healthy controls, *WM* gray matter, *WM* white matter, *IC* intracranial. Unless otherwise indicated, values are group means (SD). ^1^*t*-test, ^2^chi-square test for proportions, ^3^one-way ANCOVA controlling for total intracranial volume, ^4^Mann–Whitney *U*

### Depression and fatigue assessment

Current depression symptoms were assessed using the Hospital Anxiety and Depression Scale (HADS [38]). The Fatigue Severity Scale (FSS) was used to assess the severity and impact of fatigue on daily life [39]. For descriptive purposes, standard cutoffs on HADS and FSS were considered. A score of 8 or higher on HADS was considered as indicative of clinically significant depression symptoms [40]. A score of 36 or higher on the FSS is indicative of clinically significant perceived fatigue levels [41].

In addition, all patients received a battery of neuropsychological tests as part of the MS Epidemiology program. Results are briefly reported here to aid comparability with other studies and patient cohorts using the same or equivalent tests (e.g., [42–44]). Verbal short-term and working memory were assessed via the Memory for Digits Forward and Reverse tests from the Greek Memory Scale [45]; verbal episodic memory using the passage memory test from the Greek Memory Scale and the Rey Auditory Verbal Learning Test (AVLT) [46, 47]; semantic and phonemic verbal fluency using the verbal fluency test (SVFT and PhVFT, respectively [48]; problem solving ability via the matrix subtest from the Wechsler Adult Intelligence Scale-Fourth Edition [49]; and visuomotor processing speed and set-shifting ability via the Trail Making Test parts A and B, respectively (TMT-A and TMT-B [50]). Visuomotor coordination and manual dexterity was assessed using the Grooved Pegboard Test (Lafayette Instruments) posing similar demands as the 9 Ηole Peg test, employed by Dadar et al. [51]. For descriptive purposes, individual performance on all neuropsychological tests was converted to age- and education-adjusted standard scores using published norms developed for the native population.

### MR image acquisition and volumetry

MRI exams were performed on a 1.5T MR scanner (Vision/Sonata, Siemens, Erlangen) equipped with high-performance gradients (gradient strength: 40 mT/m; slew rate: 200 mT/m/ms) and a two-element circularly polarized head array coil (minimum voxel dimensions: 70 μm × 70 μm × 300 μm). The basic clinical imaging protocol comprised of three standard MRI sequences: (a) a 3D T1-fast spoiled gradient echo (FSPGR) (repetition time (TR) 4.98 ms, echo time (TE) 1.348 ms, voxel size 1×1×1 mm, inversion time (IT) 500 ms, acquisition time 3.50 min), (b) a 2D T2w TSE (TR/TE: 5000/98 ms, 4 mm slice thickness/1.6 mm gap, 21 oblique axial slices), and (c) a 2D T2w turbo FLAIR (TR/TE/TI: 9000/120/2600 ms, 4mm slice thickness/1.6 mm gap, 21 oblique axial and sagittal slices). Axial sections were acquired parallel to the plane connecting the anterior and posterior commissures (AC-PC line).

After the basic clinical imaging protocol, a T2*-weighted, fat-saturated 2D-FID-EPI sequence was applied for rs-fMRI, with TR 2300 ms, TE 50 ms, field of view (FOV) 192 × 192 × 108 (*x*, *y*, *z*). Acquisition voxel size was 3 × 3 × 3 mm, and whole-brain scans consisted of 36 transverse slices acquired parallel to the plane passing through the anterior and posterior commissures (AC-PC line with 3.0-mm slice thickness and no interslice gap). The scans consisted of 150 functional volumes, totaling ~6 min.

Total T2 lesion burden was performed on the T1 and FLAIR images (anonymized and compressed NIfTI files on the LesionBrain.1 pipeline) and total intracranial volume, total GM, and WM volume (normalized to the total intracranial volume after subtraction of total lesion volume) were computed using volBrain [52].

### fMRI data preprocessing and denoising

Each BOLD time series consisted of 150 dynamic volumes (the first five were ignored in all subsequent analyses). The preprocessing steps included slice time correction, realignment, segmentation of structural data, normalization into standard stereotactic Montreal Neurological Institute (MNI) space, and spatial smoothing using a Gaussian kernel of 8 mm full-width at half-maximum using SPM8. As FC is affected by head motion in the scanner, we accounted for motion artifact detection and rejection using the artifact detection tool (ART) (http://www.nitrc.org/projects/artifact detect). The first five principal components of WM and CSF regions were regressed out of the signal as well as their first-order derivatives. These steps were completed using CompCor implemented within the CONN preprocessing module [53] and executed in MATLAB. The fMRI time series were detrended and bandpass filtered in the 0.008–0.09-Hz range to eliminate low-frequency drift and high-frequency noise [34].

### Time shift analysis

For TSA calculation, only the CSF signal was regressed out of the BOLD fMRI time series, while all the remaining preprocessing steps were applied as described above (global GM and WM signals were not considered noise in the calculation of TSA [33, 34]). Firstly, a mask of the major venous sinuses was created based on the standard brain. The reference BOLD time series was calculated as the mean of all the voxel time series included in the venous mask. Then, voxel-wise cross-correlations were calculated in reference to this regressor for lags of −3 TRs to 3 TRs (or −6.96 to 6.96 s). This entails the computation of the lagged versions of each voxel time series (−3 TR to 3 TR) and of the correlation coefficient of each lagged version of the time series with the reference signal. The lag value corresponding to the highest correlation coefficient was then assigned to each voxel as its time shift value.

### Voxel-wise FC and ROI-based connectivity

Voxel-wise global connectivity was assessed through the intrinsic connectivity contrast (ICC), which is based on the graph theoretical measure of degree. Degree signifies the number of other nodes connected to each node, while the calculation of ICC, on a weighted graph, considers the connectivity strengths of all connections present for each node. Specifically, a voxel’s ICC value is computed as the mean of that voxel’s time series correlation values with all other voxels’ time series, squared, and therefore reflects the degree of association between the time series of a given voxel with all the remaining brain voxels. In the present analysis, only gray matter voxels were considered by applying a mask consisting of all 90 regions included in the automated anatomical labelling atlas (AAL [54]).

Graph theory modeling of whole-brain connectivity was also performed to compute the nodal metrics of betweenness centrality and local efficiency indices for each AAL area where significant correlations were found between fatigue (or depression) scores and ICC or TSA values. Regional connectivity was computed via in-house MATLAB scripts, and regional graph metrics were computed using the Brain Connectivity Toolbox (BCT) (sites.google.com/site/bctnet/). Implementation details can also be found at: github.com/njsimos/sfcg_thresh_graph_metrics.

### Connectivity–hemodynamic coupling

Coupling was indexed by the percentage of voxels within each AAL region displaying relatively high ICC and hemodynamic lead or low ICC and hemodynamic lag. Uncoupling was indexed by the percentage of voxels displaying high ICC and hemodynamic lag or low ICC and hemodynamic lead. These indices were derived from four whole-brain voxel-wise overlay masks that were independently computed. Two maps were calculated of voxels displaying coupling between hemodynamics and intrinsic connectivity (i.e., one map of voxels displaying relatively high global connectivity and hemodynamic lead and a second map comprising voxels displaying low connectivity and hemodynamic lag). Two additional maps of voxels displaying uncoupling were derived (i.e., relatively high connectivity and hemodynamic lag or relatively low connectivity and hemodynamic lead). High FC was defined as a significantly high (or low, respectively) ICC value in whole-brain, voxel-wise analyses (at *p*<.001 uncorrected). Hemodynamic lead of a given voxel was indicated by TSA values < −1 TR, whereas hemodynamic lag was indicated by TSA values > 1 TR. The percentage of voxels within each AAL region displaying coupling or uncoupling according to the aforementioned definitions served as the dependent variables in these analyses. All ICC, TSA, and coupling indices were computed via custom scripts in MATLAB. Code for all analyses is publicly available from the authors (github.com/njsimos/tsa_icc).

### Regions of interest

ICC, TSA, and ICC-TSA overlay masks were computed for 50 AAL regions given their possible role for depression and/or fatigue (see Supplementary Material 1). The definition of the depression-related brain regions was based on recent meta-analyses of studies comparing people with depression and healthy controls in resting-state and task-based fMRI, and GM morphometry [55–57]. The definition of the fatigue-related brain regions was based on a recent meta-analysis of studies comparing people with MS experiencing high vs. low levels of fatigue in gray matter volumetry [58] and recent research papers comparing the two groups in resting-state FC metrics [23, 25, 59].

### Statistical analysis

#### Volumetric analyses

The two patient groups were compared on total intracranial volume using one-way ANOVA and on total GM, total WM, and total T2 lesion volumes using one-way ANCOVAs, controlling for total intracranial volume.

#### fMRI analyses

Voxel-wise analyses using SPM12 were conducted to identify brain regions where individual differences in hemodynamics or FC correlated with the severity of fatigue or depression symptoms in each patient group. Analyses were restricted to the GM voxels included in the 50 predefined AAL ROIs (see Supplementary Table 1). In two separate sets of models, the covariate of interest consisted of (a) FSS scores computed by regressing out HADS depression subscale scores and (b) HADS depression subscale scores computed by regressing out FSS scores. Total intracranial volume served as a nuisance variable. Results were thresholded at *p*<.01 uncorrected given the restricted set of tests performed.

Associations of FSS and/or HADS scores (as defined above) with nodal FC indices (regional betweenness centrality and local efficiency) and with FC hemodynamics coupling indices (percentage of voxels displaying coupling or uncoupling between ICC and TSA values) were also performed. Partial correlations were computed controlling for total intracranial volume evaluated at *p*<.01. These analyses were conducted in SPSS version 20 (SPSS Inc., Chicago, IL, USA).

## Results

### Cognitive and emotional status of CIS and RR-MS patients

As expected, the frequency of patients reporting clinically significant depression-related symptoms was higher in the RR-MS (32.0%) than in the CIS group (8.0%; *p*=.05). Similarly, the patients scoring over 36 points on FSS, indicating significant burden of fatigue, was very high among RR-MS patients (69.0%) and substantially lower in the CIS group (33.3%; *p*=.014). Comorbidity of fatigue and depression symptoms was higher in the RR-MS (27.6%) than in the CIS group (4.1%; *p*=.03) (Table 2). Symptom severity did not correlate with cerebral WM or GM volume or with total lesion volume (*p*>.2). On average, patients in both groups performed within normal range on fluid intelligence, immediate and delayed verbal episodic memory, and semantic verbal fluency. As also expected, performance on tasks placing significant demands on manual motor control and processing speed (Trail Making Test (TMT) A and B, Grooved Pegboard) was significantly lower among RR-MS as compared to CIS patients (*p*=.02 to *p*=.001; Table 2). On average, patients with RR-MS scored one standard deviation (SD) below the age- and education-adjusted population mean on phonemic verbal fluency, TMT-A, and TMT-B, and 5 SDs below the expected mean on the Grooved Pegboard Test.

**Table 2:** Cognitive and emotional status of CIS and RR-MS patients

	CIS (*n*=24)		RR-MS (*n*=29)		*p* value
	Raw score	*z* score	Raw score	*z* score	
AVLT trials 1–5	55.6 (10.5)	0.21 (1.1)	54.2 (10.2)	−0.12 (1.2)	0.4^1^
AVLT trial 7	12.3 (2.3)	0.58 (1.0)	11.6 (3.6)	0.13 (1.3)	0.2^1^
AVLT retention	0.95 (0.1)	0.92 (0.6)	0.89 (0.1)	0.30 (0.9)	0.03^1^
Digit span	14.3 (2.6)	−0.45 (0.3)	13.9 (3.1)	−0.62 (0.4)	0.3^1^
SVFT	50.0 (10.8)	−0.13 (1.0)	48.3 (9.5)	−0.48 (0.9)	0.2^1^
PhVFT	28.2 (10.3)	−0.63 (1.0)	26.6 (10.2)	−1.00 (0.8)	0.1^1^
TMT-A (s)	28.9 (9.3)	0.09 (0.6)	41.0 (29.3)	−0.83 (1.8)	0.02^1^
TMT-B (s)	64.9 (35.5)	−0.11 (1.9)	95.2 (70.0)	−1.22 (2.4)	0.04^1^
Pegboard (s)	70.6 (9.1)	0.66 (1.2)	104.8 (47.8)	−5.24 (6.2)	0.001^1^
WAIS-IV matrices	21.3 (7.4)	0.29 (1.1)	20.0 (6.8)	−0.21 (1.0)	0.4^1^
HADS depression	4.6 (3.1)	-	5.8 (2.6)	-	0.1^1^
% >8 points	8.3%	-	31.0%	-	0.05^2^
FSS	33.1 (15.9)	-	42.6 (15.0)	-	0.03^1^
% >36 points	33.3%	-	69.0%	-	0.01^2^

Unless otherwise indicated, values correspond to mean (SD). *Z* scores: age- and education-adjusted based on population norms; *AVLT* auditory verbal learning test; *SVFT* semantic verbal fluency test; *PhVFT* phonemic verbal fluency test; *TMT* Trail Making Test; *WAIS-IV* Wechsler Adult Intelligence Scale, Fourth Edition; *FSS* Fatigue Severity Scale; *HADS* Hospital Anxiety and Depression Scale. ^1^One-way ANOVA

### Volumetric analyses

The two patient groups were comparable on total intracranial volume (*p*=.5), and on total, GM volume adjusted for total intracranial volume (*p*=.5), although RR-MS patients displayed larger total T2 lesion volumes (*p*=.03) and marginally lower total WM volumes than both CIS patients (*p*=.07) and healthy controls (*p*=.06; see Table 1). As expected, the RR-MS group displayed significantly higher lesion burden than the CIS group (*p*=.03).

### Hemodynamics and connectivity measures uniquely associated with fatigue

**Fig. 1:**
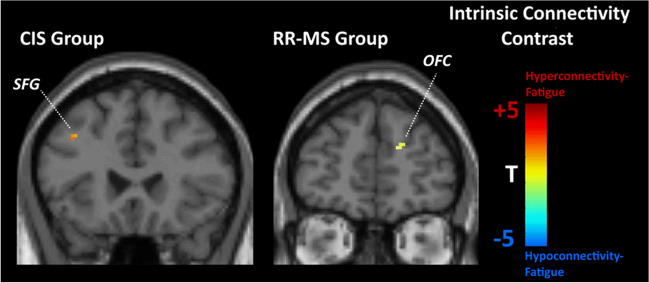
Statistical parametric map of correlations between voxel-level intrinsic connectivity contrast (ICC) values and Fatigue Severity Scale score (FSS) (controlling for depression symptom severity) among patients with relapsing–remitting multiple sclerosis (RR-MS) and clinically isolated syndrome (CIS). Voxels shown are significant at *p*<.001, uncorrected with minimum cluster size of 20 voxels. Abbreviations: OFC, orbitofrontal cortex (Montreal Neurological Institute (MNI): 13, 49, 21); SFG, superior frontal gyrus (MNI: −39, 23, 38)

**Fig. 2:**
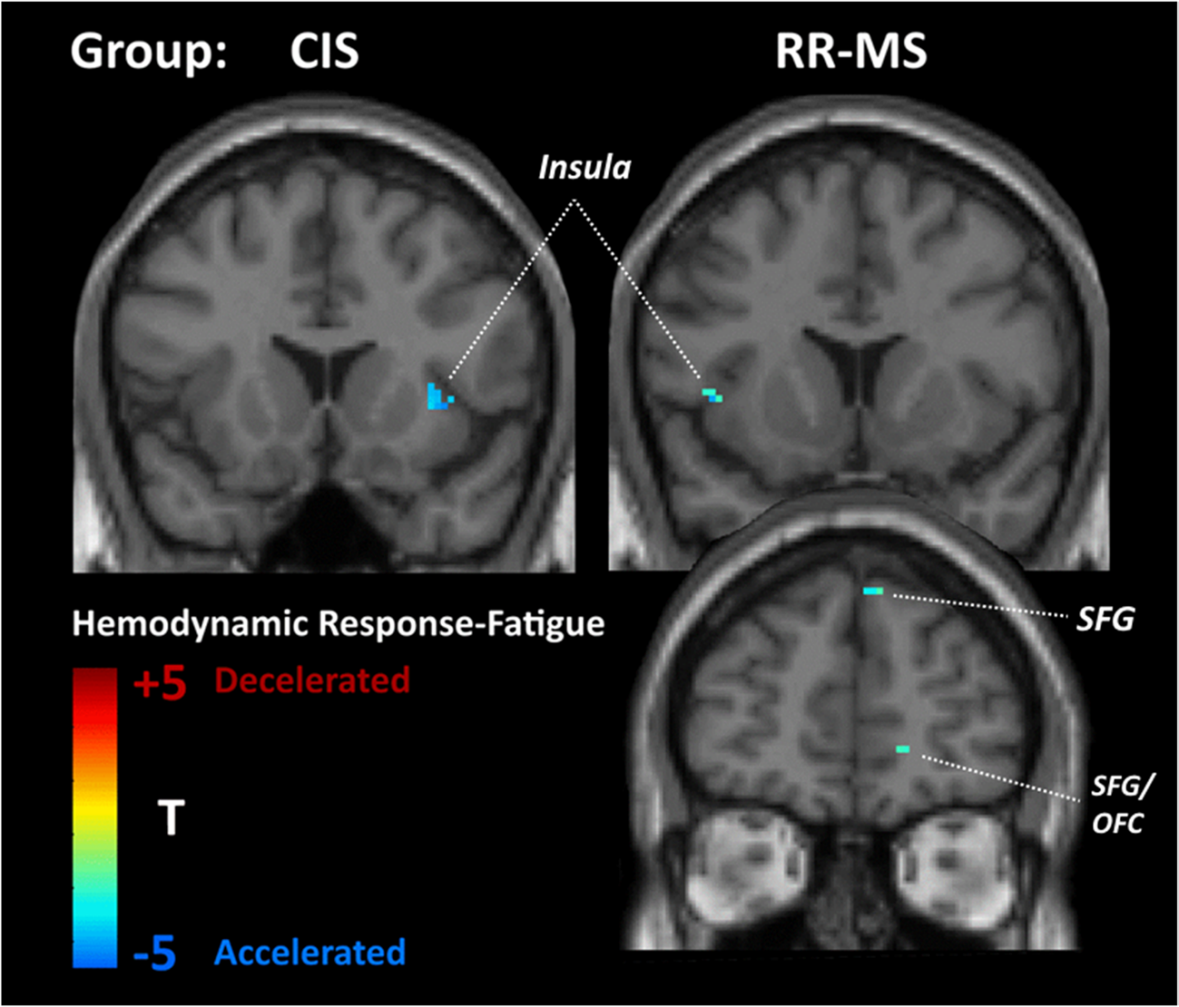
Statistical parametric map of correlations between voxel-level estimates of hemodynamic timing and FSS scores (controlling for depression symptom severity) among patients with RR-MS and CIS. Voxels shown are significant at *p*<.001, uncorrected with minimum cluster size of 20 voxels. MNI coordinates for the right insula were 33, 16, 2 and for the left insula −40, 16, 5. Abbreviations: OFC, orbitofrontal cortex (MNI: 11, 51, −1); SFG, superior frontal gyrus (MNI: 8, 51, 42).

In CIS patients, fatigue, after controlling for depression symptoms, was associated (at *p*<.001 uncorrected) with *hyperconnectivity* in the medial portion of the superior frontal gyrus (SFG) (BA 8 in the left hemisphere; see Fig. 1). With respect to hemodynamic status, there was accelerated hemodynamic response in the right insula (see Fig. 2).

In the RR-MS group, severity of fatigue, after controlling for depression symptoms, was associated with *heightened role* of the left amygdala within the entire brain network (indicated by a marginally significant positive association of betweenness centrality of this region with fatigue ratings: *r*=.561, *p*=.002). Additionally, there was *hypoconnectivity* of the right orbitofrontal cortex (OFC) (BA 10; see Fig. 1). These effects were paralleled by accelerated hemodynamic response in the left insula and medial portion of the SFG (BA 8 in the right hemisphere; see Fig. 2).

**Fig. 3:**
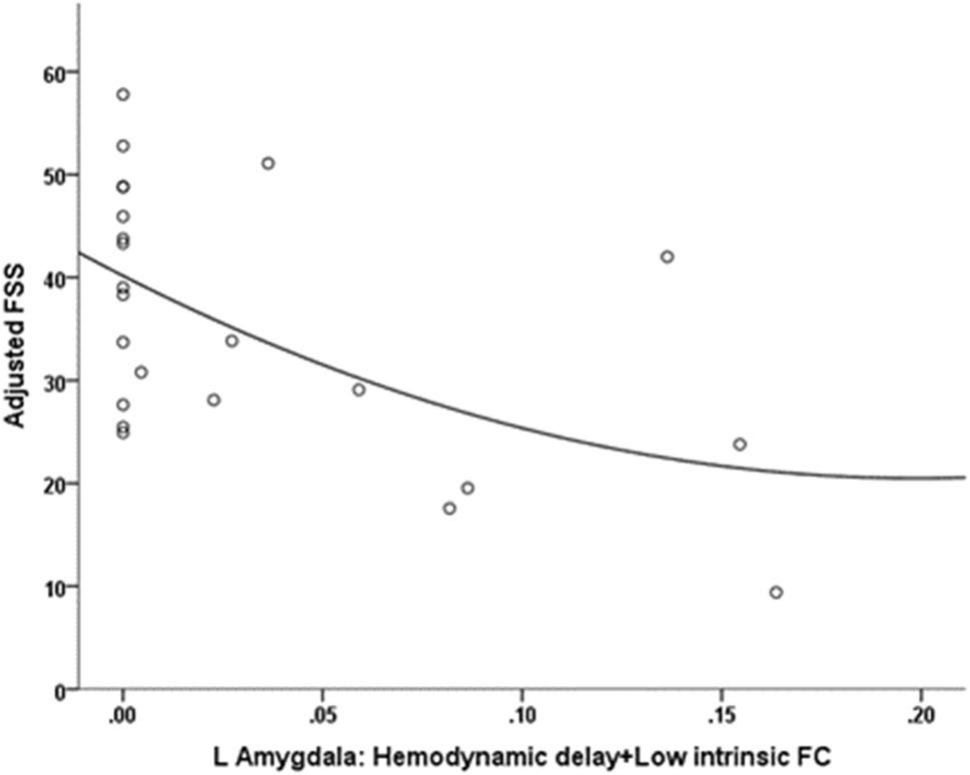
Association between FSS score adjusted for depression symptom severity and the proportion of voxels displaying hemodynamic delay coupled with low intrinsic functional connectivity (FC) in the left amygdala (automated anatomical labelling atlas (AAL) region 41, MNI: −23, −1, −17) among patients with CIS (*r*=−.548).

In the CIS group, fatigue was associated with *reduced coupling* in the left amygdala, as indicated by lower percentage of voxels displaying hemodynamic delay (>1 TR) coupled with low intrinsic connectivity (*r*=−.548, *p*=.007; see Fig. 3). This effect remained significant (*p*<.01) after controlling for total GM volume and total lesion volume. In order to explore this result further, we compared CIS patients with the group of healthy volunteers. Given that only 8 CIS patients had FSS scores > 36 (clinically validated cutoff for presence of significant fatigue), we used the group median score (32 points) to assign CIS patients to a low (*n*=12) and a high fatigue group (*n*=12). We found that the degree of coupling among CIS patients who experienced higher levels of fatigue was significantly lower than that observed among age- and sex-matched healthy volunteers (*p*<.01) and marginally lower than in non-fatigued CIS patients (*p*=.07). In the RR-MS group, associations between fatigue and coupling indices did not reach significance (*p*>.1).

### Hemodynamics and intrinsic connectivity uniquely associated with depression

**Fig. 4:**
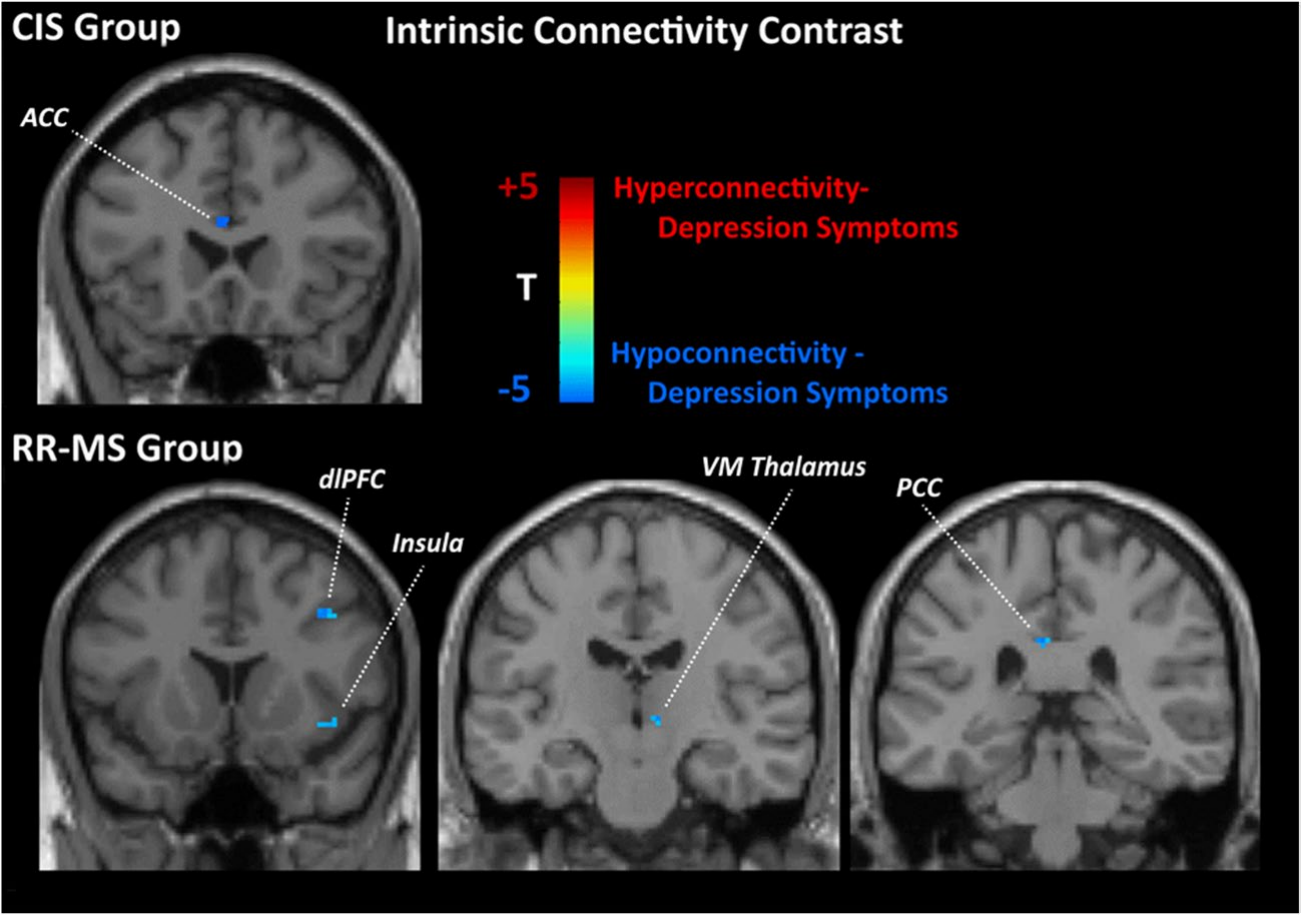
Statistical parametric map of correlations between voxel-level intrinsic connectivity contrast (ICC) values and Hospital Anxiety and Depression Scale (HADS) depression subscale scores (controlling for severity of fatigue) among patients with RR-MS and CIS. Voxels shown are significant at *p*<.001, uncorrected with minimum cluster size of 20 voxels. MNI coordinates for the right insula were 42, 15, −5 and for the ventromedial thalamus 7, −16, 2. Abbreviations: VM, ventromedial; dlPFC, dorsolateral prefrontal cortex (MNI: 47, 18, 31); ACC, anterior cingulate cortex (MNI: −1, 23, 22); PCC, posterior cingulate cortex (MNI: −3, −32, 33)

**Fig. 5:**
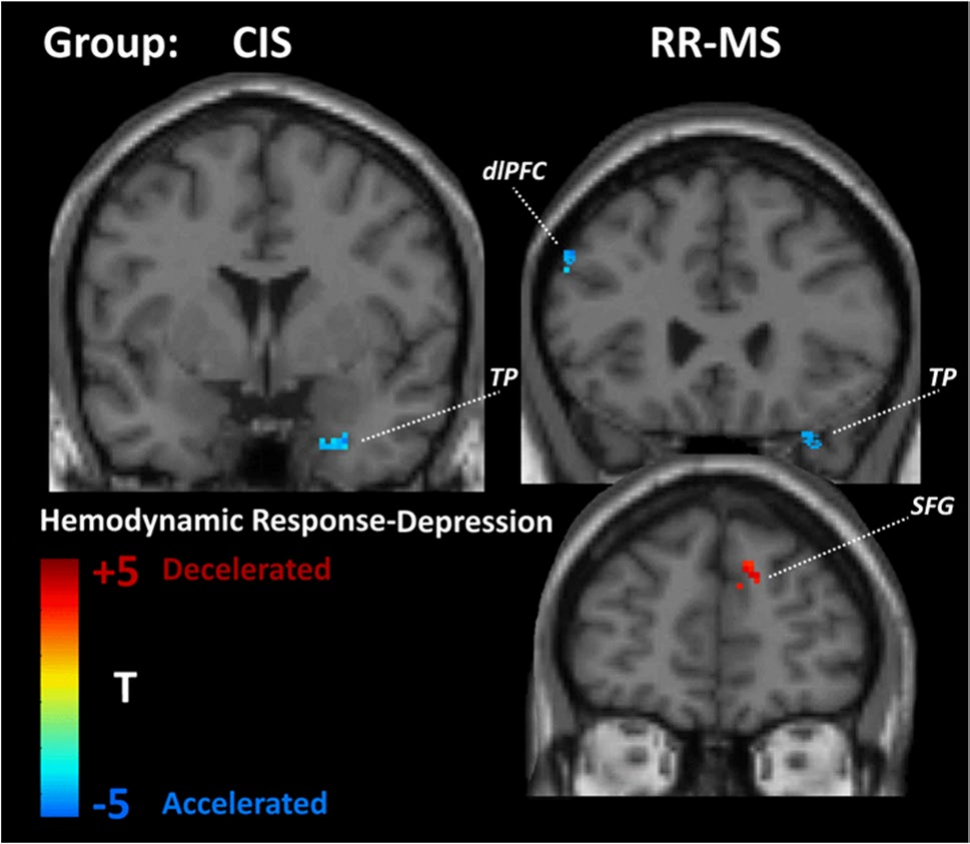
Statistical parametric map of correlations between voxel-level estimates of hemodynamic timing and HADS depression subscale scores (controlling for severity of fatigue) among patients with RR-MS and CIS. Voxels shown are significant at *p*<.001, uncorrected with minimum cluster size of 20 voxels. Abbreviations: TP, temporal pole (MNI: 25, 2, −33 (CIS group), 37, 25, −29 (RR-MS group)); SFG, superior frontal gyrus (MNI: 10, 49, 32); dlPFC, dorsolateral prefrontal cortex (MNI: −50, 26, 33)

In the CIS group, depression symptom severity was associated with *hypoconnectivity* of the dorsal anterior cingulate cortex (ACC) (see Fig. 4) and accelerated hemodynamic response in the right limbic temporal pole (see Fig. 5). In addition, depression symptom severity in RR-MS patients was associated with *hypoconnectivity* of the insula, ventromedial thalamus, and dorsolateral prefrontal cortex (dlPFC) (BA 9) in the right hemisphere and of the left posterior cingulate cortex (PCC) (see Fig. 4). These effects were paralleled by decelerated hemodynamic response in the medial portion of the SFG in the right hemisphere and accelerated hemodynamic response in the right limbic temporal pole and left dlPFC (see Fig. 5).

**Fig. 6:**
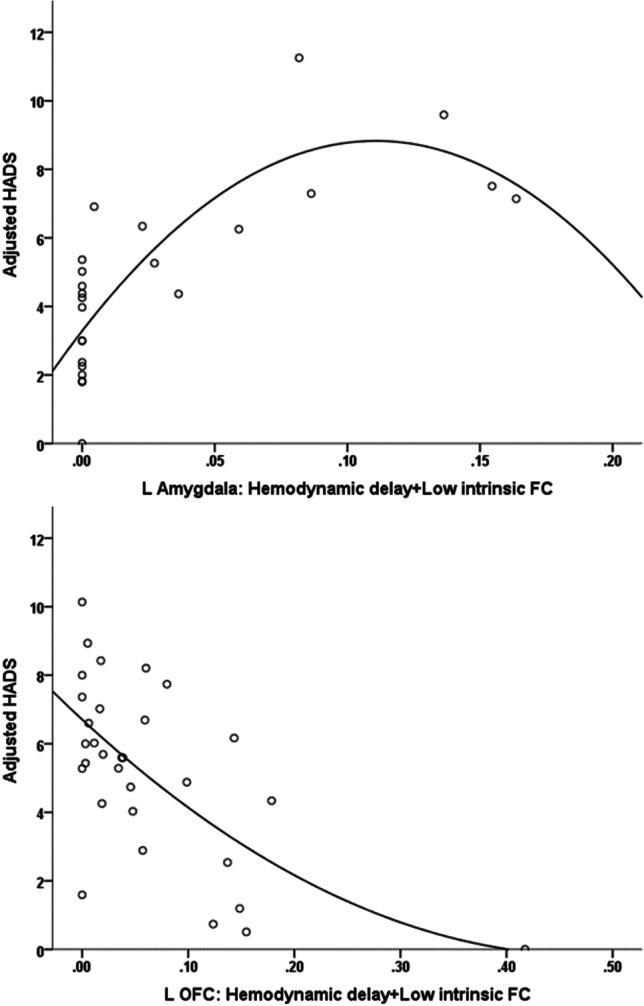
Association between HADS depression subscale scores (adjusted for fatigue self-ratings) and the proportion of voxels displaying hemodynamic delay coupled with low intrinsic functional connectivity (FC) in the left amygdala (AAL region 41, MNI: −23, −1, −17) among patients with CIS (*r*=.799) and in the left superior orbitofrontal cortex (OFC) (AAL region 5, MNI: −17, 47, −13) among RR-MS patients (*r*=−.560)

Among patients with CIS, depression symptom severity was associated with *increased coupling* in the left amygdala, as indicated by higher percentage of voxels displaying hemodynamic delay (>1 TR) coupled with low intrinsic connectivity (*r*=.799, *p*<.001; see Fig. 6). This effect remained significant (*p*<.001) after controlling for total GM volume and total lesion volume. Moreover, the degree of coupling among CIS patients who experienced significant depression symptomatology was marginally higher than that observed in the healthy control group (*p*=.02) and marginally lower than non-depressed CIS patients (*p*=.08). The correlation of amygdala volume with depression severity, controlling for total intracranial volume, did not reach significance (|*r*|<.39, *p* >.1).

In the RR-MS group, depression symptom severity was associated with *reduced coupling* in the medial orbitofrontal part of the left SFG as indicated by fewer voxels displaying hemodynamic delay and low intrinsic connectivity in this region (*r*=−.560, *p*=.001; see Fig. 6). Compared to the healthy controls, the subgroup of potentially depressed RR-MS patients displayed marginally greater proportion of voxels with hemodynamic delay coupled with hypoconnectivity (*p*=.03), whereas the difference between depressed and non-depressed RR-MS patients approached significance (*p*=.08).

## Discussion

Despite several reports on the neuroimaging correlates of fatigue and depression in RR-MS patients, there is virtually no data regarding FC or hemodynamic status in CIS. The main findings of the current study can be summarized as follows: (a) CIS patients are characterized by aberrant hemodynamic and FC abnormalities in specific brain areas linked to fatigue (left amygdala, insula, medial SFG) or to depression (left amygdala, ACC, right temporal pole); (b) the topography, direction, and magnitude of these alterations in CIS patients are different from RR-MS patients and; and (c) there is inverse correlation between fatigue and depression with hemodynamic connectivity coupling in left amygdala in CIS patients.

### Fatigue vs depression at different stages of MS: the role of the left amygdala and insular cortex

In CIS, higher fatigue levels are associated with a relatively smaller portion of the *left amygdala* displaying reduced FC coupled with hemodynamic delay, while the left amygdala volume was significantly smaller in highly fatigued patients as compared to age- and sex-matched healthy volunteers. We surmise that relatively high levels of hemodynamic lag combined with hypoconnectivity represent baseline amygdala function (i.e., *at low fatigue levels*). Conversely, reduced hemodynamic lag combined with hypoconnectivity within the same section of the amygdala is consistent, albeit, indirectly, with previous evidence of regional hyperperfusion in CIS [5]. In RR-MS patients, however, fatigue symptoms were related to a relatively stronger role of the *left amygdala* within the whole-brain FC network (as indexed by betweenness centrality) regardless of hemodynamic status (to the extent indexed by TSA). Reports implicating aberrant amygdala function in fatigue have been scarce and overshadowed by findings of amygdala hyper- or hypoconnectivity in emotional and anxiety disorders [34]. Nevertheless, two recent diffusion tensor imaging (DTI) studies [25, 60] have reported abnormal structural integrity in the amygdala and interconnecting fiber tracts in relation to fatigue in MS, supporting the notion that both structural and functional integrities of the amygdala are crucial for triggering the subjective experience of fatigue in MS patients. Taken together, these findings may imply that the amygdala retains its central role within the circuit related to fatigue symptoms as the disease progresses despite possible damage in surrounding white matter.

In sharp contrast to fatigue in CIS, *depression was related to increased hemodynamic connectivity coupling in the left amygdala*: patients who reported more severe depression symptomatology presented with significantly higher percentage of voxels displaying hemodynamic delay coupled with low ICC compared to both non-depressed CIS patients and healthy controls. This finding was corroborated by an opposite trend involving the percentage of voxels displaying hemodynamic lead coupled by higher intrinsic hyperconnectivity. Taken together, these findings are indicative of reduced neural functionality of the left amygdala being involved in depression symptom severity in this group in agreement with previous studies in MS patients [61]. It should be noted that the evidence regarding amygdala resting-state connectivity in relation to primary depressive illnesses is mixed [62]. Given that this trend is in the opposite direction than the one found for fatigue, it appears that in CIS patients, the type of prevailing subjective symptom experienced by each patient may depend upon individual differences in the balance between hemodynamic activity and neuronal communication of the amygdala with other brain structures.

### Fatigue–depression dissociations in insular and prefrontal hemodynamics

Fatigue was also found to correlate positively with hemodynamic lead in the insula (in both CIS and RR-MS patients). This finding is in partial agreement with structural [25] and FC findings [26, 59]. Both the amygdala and insular cortex play important roles in interception, serving as key targets of afferent nociceptive signals traveling along the vagus nerve [63]. Taken together these findings are consistent with the notion that structurally and functionally intact insular cortex and amygdala are crucial for the experience of fatigue in MS.

Conversely, depression symptom severity was positively related to hypoconnectivity of the insula in patients with RR-MS. One possible explanation is that accelerated hemodynamic response in this structure is linked to heightened attention toward internal somatic symptoms which accompany fatigue [26], whereas insula hypoconnectivity is linked to anxious avoidance manifestations which are measured (directly or indirectly) by brief rating scales of depression, such as the HADS [64]. Moreover, the density of hyperintense lesions in the white matter adjacent to insular cortex correlated significantly with depression symptom severity in MS patients [65].

Another brain region where a clear dissociation in hemodynamic correlates of fatigue vs. depression symptom severity was found was the medial portion of the right SFG in the RR-MS group: whereas fatigue severity was positively associated with increased hemodynamic lead, depression symptom severity was positively related to increased hemodynamic lag.

### Additional neuroimaging correlates of fatigue and depression in CIS and RR-MS

Fatigue severity was positively associated with *hyperconnectivity* of medial SFG (BA 8) in the CIS group and *hypoconnectivity* of dorsal OFC (BA 10) in RR-MS patients. Reduced FC in this region in RR-MS is consistent with tractography findings of a negative correlation between FA in the corona radiata and superior longitudinal fasciculus and fatigue symptoms in this group [27, 66].

Regarding depression, symptom severity was positively related to *hypoconnectivity* of the dlPFC (BA 9) and increased proportion of voxels displaying *hemodynamic lag coupled with hypoconnectivity* within superior OFC in RR-MS patients. Taken together, reduced functionality of dorsal prefrontal areas in depression is consistent with evidence of positive associations between prefrontal atrophy and depression in MS [67, 68].

Further, depression was associated with hypoconnectivity of the dorsal ACC in CIS. Earlier findings that ACC hypoconnectivity was associated with higher fatigue ratings in MS patients [59] may, thus, be attributed to the confounding effects of subclinical, comorbid depression symptoms [69]. This notion is supported by the association between ACC atrophy and depression symptom severity reported by Gobbi et al. [67].

We also found evidence that depression symptom severity correlated with thalamic hypoconnectivity in RR-MS, which is consistent with the reported link between thalamic atrophy and depression [70]. Thus, reports of a negative correlation between connectivity [71] and microstructural changes in the thalamus [72] with fatigue may be at least in part related to the confounding effect of subclinical depression symptoms as also suggested by Palotai et al. [73] on the basis of volumetric lesion analyses in MS patients.

Finally, a common finding to both groups of patients is that depression symptom severity was related to accelerated hemodynamic response of the right temporal pole. Increased hemodynamic reactivity is consistent with some evidence of increased FC of temporal limbic structures at rest in relation to depression and anxiety symptoms [74].

### Study limitations and further considerations

There are important limitations in this study. Firstly, direct associations between emotional status and brain function were only assessed among patients. Hence, we cannot conclude that the reported associations are specific to the putative brain pathophysiology characteristic of MS or, alternatively, that they reflect the combined direct impact of the disease on brain function and its indirect effects on emotional well-being through the physical burden incurred by the disease. Comparing subgroups of patients formed on the basis of fatigue or depression symptom severity provides some support to the claim that results may be specific to fatigue or depression, respectively, although the size of the subgroups in the present study was insufficient to draw firm conclusions. Properly addressing this hypothesis requires direct comparisons of MS/CIS patients with healthy controls, although in the latter, group associations between brain activity indices and symptom severity are not likely to produce significant results due to insufficient between-person variability and restricted range of scores on the respective self-report scales.

It should further be noted that a more direct test of the independent (or perhaps additive) effect of fatigue and depression on patterns of BOLD oscillations entails comparing groups of patients formed on the severity of respective symptoms (i.e., patients with predominant depression symptoms and patients with predominant fatigue symptoms). Additional subgroups of patients who manifested significant depression and fatigue symptoms would help clarify whether the effects of each type of symptom are additive or multiplicative (implying some form of interaction between the respective neural substrates). This approach would, however, require a much larger cohort of patients than those available in the present study. Larger samples of RR-MS and CIS patients would also permit setting more strict statistical thresholds in both whole-brain and ROI-based analyses.

Moreover, hemodynamic status was assessed based on time lags (or leads) in the resting-state time series across brain voxels. These measures of time shift in the phase of low-frequency BOLD oscillations are presumed to reflect hemodynamic transfer lag or lead times and, at least, in part serve as indirect indices of regional brain perfusion [75, 76]. Indices of hemodynamic lag have been validated in cases of severe acute or chronic brain ischemia due to large vessel occlusion [28–32] or milder widespread hemodynamic deficits in patients with Alzheimer’s disease [33] and NPSLE [34, 35] and have been shown to represent hypoperfused tissue. On the contrary, the significance of hemodynamic lead has not been explored systematically.

Technical limitations of the fMRI data should also be considered, and the potential benefits from increased temporal and spatial resolution by using longer BOLD time series obtained in a 3T scanner are worth mentioning. We attempted to mitigate these limitations by implementing an ROI-based approach, which does not make claims of fine-grained connectivity maps. However, increased signal-to-noise ratio and especially longer time series achieved by lower TR values could potentially benefit the analysis.

Finally, interpretation of the current results should consider that patients included in the CIS group had experienced a single neurological event, on average 4 years prior to inclusion in the study. Although they did not show evidence of motor or cognitive impairment and had a very low rate of clinically significant depression symptoms (8%), they reported clinically significant symptoms of fatigue at a relatively high rate (33%). Patients who either experienced a second neurological episode or presented with dissemination in space meeting the revised McDonald criteria for MS diagnosis had longer disease course and were three times as likely to report clinically significant depression symptoms and twice as likely to experience significant fatigue than patients in the CIS group.

## Conclusion

In the current study, we used rs-fMRI and time shift analysis for the concurrent estimation of FC and hemodynamics and hemodynamic connectivity coupling in CIS and RR-MS patients. We documented distinct FC and hemodynamic features, related to fatigue and depression symptomatology even among CIS patients. These changes mainly involved the left amygdala, insula, and prefrontal areas and differed considerably between CIS and RR-MS groups. At the patient level, associations between neuroimaging features and severity of fatigue or depression symptoms were in some cases in opposite directions, indicating dependence of the prevailing subjective symptoms on individual differences in the balance between hemodynamics and FC in these brain structures.

## Supplementary information

ESM 1 (DOCX 72 kb)

## Data Availability

The computed metrics derived from resting-state fMRI recordings will be available upon request.
