# Supplementary material for: Distinct hemodynamic and functional connectivity features of fatigue in clinically isolated syndrome and multiple sclerosis: accounting for the confounding effect of concurrent depression symptoms
Source: Neuroradiology. 2023 Jun 10;65(8):1287–300. doi: 10.1007/s00234-023-03174-1 (PMC10338578; doi:10.1007/s00234-023-03174-1)
Supplement: Supplementary file 1 — (DOCX 72 kb) [file 234_2023_3174_MOESM1_ESM.docx]

Supplementary Table 1. Regions of interest included in the analyses

| Labels | Aal name | Regions | #. |
| --- | --- | --- | --- |
| 3 | Frontal_Sup_L | Superior frontal gyrus, dorsolateral | 1 |
| 4 | Frontal_Sup_R | Superior frontal gyrus, dorsolateral | 2 |
| 5 | Frontal_Sup_Orb_L | Superior frontal gyrus, orbital part | 3 |
| 6 | Frontal_Sup_Orb_R | Superior frontal gyrus, orbital part | 4 |
| 9 | Frontal_Mid_Orb_L | Middle frontal gyrus, orbital part | 5 |
| 10 | Frontal_Mid_Orb_R | Middle frontal gyrus, orbital part | 6 |
| 23 | Frontal_Sup_Medial_L | Superior frontal gyrus, medial | 7 |
| 24 | Frontal_Sup_Medial_R | Superior frontal gyrus, medial | 8 |
| 25 | Frontal_Mid_Orb_L | Superior frontal gyrus, medial orbital | 9 |
| 26 | Frontal_Mid_Orb_R | Superior frontal gyrus, medial orbital | 10 |
| 29 | Insula_L | Insula | 11 |
| 30 | Insula_R | Insula | 12 |
| 31 | Cingulum_Ant_L | Anterior cingulate and paracingulate gyri | 13 |
| 32 | Cingulum_Ant_R | Anterior cingulate and paracingulate gyri | 14 |
| 33 | Cingulum_Mid_L | Median cingulate and paracingulate gyri | 15 |
| 34 | Cingulum_Mid_R | Median cingulate and paracingulate gyri | 16 |
| 35 | Cingulum_Post_L | Posterior cingulate gyrus | 17 |
| 36 | Cingulum_Post_R | Posterior cingulate gyrus | 18 |
| 37 | Hippocampus_L | Hippocampus | 19 |
| 38 | Hippocampus_R | Hippocampus | 20 |
| 39 | ParaHippocampal_L | Parahippocampal gyrus | 21 |
| 40 | ParaHippocampal_R | Parahippocampal gyrus | 22 |
| 41 | Amygdala_L | Amygdala | 23 |
| 42 | Amygdala_R | Amygdala | 24 |
| 45 | Cuneus_L | Cuneus | 25 |
| 46 | Cuneus_R | Cuneus | 26 |
| 65 | Angular_L | Angular gyrus | 27 |
| 66 | Angular_R | Angular gyrus | 28 |
| 67 | Precuneus_L | Precuneus | 29 |
| 68 | Precuneus_R | Precuneus | 30 |
| 69 | Paracentral_Lobule_L | Paracentral lobule | 31 |
| 70 | Paracentral_Lobule_R | Paracentral lobule | 32 |
| 71 | Caudate_L | Caudate nucleus | 33 |
| 72 | Caudate_R | Caudate nucleus | 34 |
| 73 | Putamen_L | Lenticular nucleus, putamen | 35 |
| 74 | Putamen_R | Lenticular nucleus, putamen | 36 |
| 75 | Pallidum_L | Lenticular nucleus, pallidum | 37 |
| 76 | Pallidum_R | Lenticular nucleus, pallidum | 38 |
| 77 | Thalamus_L | Thalamus | 39 |
| 78 | Thalamus_R | Thalamus | 40 |
| 81 | Temporal_Sup_L | Superior temporal gyrus | 41 |
| 82 | Temporal_Sup_R | Superior temporal gyrus | 42 |
| 83 | Temporal_Pole_Sup_L | Temporal pole: superior temporal gyrus | 43 |
| 84 | Temporal_Pole_Sup_R | Temporal pole: superior temporal gyrus | 44 |
| 85 | Temporal_Mid_L | Middle temporal gyrus | 45 |
| 86 | Temporal_Mid_R | Middle temporal gyrus | 46 |
| 87 | Temporal_Pole_Mid_L | Temporal pole: middle temporal gyrus | 47 |
| 88 | Temporal_Pole_Mid_R | Temporal pole: middle temporal gyrus | 48 |
| 89 | Temporal_Inf_L | Inferior temporal gyrus | 49 |
| 90 | Temporal_Inf_R | Inferior temporal gyrus | 50 |
